# Therapeutic antitumor efficacy of anti-epidermal growth factor receptor antibody, cetuximab, against malignant pleural mesothelioma

**DOI:** 10.3892/ijo.2012.1607

**Published:** 2012-08-24

**Authors:** JUN KURAI, HIROKI CHIKUMI, KIYOSHI HASHIMOTO, MIYAKO TAKATA, TAKANORI SAKO, KOSUKE YAMAGUCHI, NAOKI KINOSHITA, MASANARI WATANABE, HIROKAZU TOUGE, HARUHIKO MAKINO, TADASHI IGISHI, HIRONOBU HAMADA, SEIJI YANO, EIJI SHIMIZU

**Affiliations:** 1 Division of Medical Oncology and Molecular Respirology, Department of Multidisciplinary Internal Medicine, Faculty of Medicine, Tottori University, Yonago-shi, Tottori-ken 683-8504;; 2 Graduate School of Health Sciences, Hiroshima University, Minami-ku, Hiroshima 734-8553;; 3 Division of Medical Oncology, Cancer Research Institute, Kanazawa University, Kanazawa, Ishikawa 920-0934, Japan

**Keywords:** malignant pleural mesothelioma, cetuximab, antibody-dependent cellular cytotoxicity, intrathoracic therapy

## Abstract

Epidermal growth factor receptor (EGFR) is commonly overexpressed in malignant pleural mesothelioma (MPM). Cetuximab is a chimeric mouse-human antibody targeted against EGFR and induces potent antibody-dependent cellular cytotoxicity (ADCC). The action of cetuximab against MPM cells has not been well studied. Therefore, in this study, we investigated the antitumor activity of cetuximab against MPM cell lines, particularly with respect to ADCC activity *in vitro* and *in vivo*. EGFR expression of MPM cells was measured by a quantitative flow cytometric analysis and immunohistochemistry. The effect of cetuximab on growth inhibition was assessed using a modified MTT assay. The ADCC activity was measured by a 4-h ^51^Cr release assay using fresh or IL-2-activated peripheral blood mononuclear cells. *In vivo* antitumor activity of cetuximab was evaluated using an orthotopic implantation mouse model. Cetuximab-mediated ADCC activity against MPM cells was observed at low concentration (0.25 mg/ml) and was enhanced by IL-2, whereas no direct effect on growth inhibition was detected. A logarithmic correlation was observed between the number of EGFRs on MPM cells and ADCC activity. Low EGFR expression on the MPM cells, which was weakly detectable by immunohistochemistry, was sufficient for maximum ADCC activity. In the mouse model, cetuximab treatment with or without IL-2 significantly inhibited intrathoracic tumor growth and prolonged their survival. Our study shows that cetuximab has potent anti-MPM activity both *in vitro* and *in vivo*, mainly through the immunologic mechanism of ADCC. Cetuximab has the potential to be used as a novel therapy for MPM patients.

## Introduction

Malignant pleural mesothelioma (MPM) is a rare and highly aggressive neoplasm, which arises from the pleural, pericardial, or peritoneal lining. Most patients with MPM have a history of exposure to carcinogenic asbestos fibers (1,2), particularly those of the amphibole type, or to naturally occurring erionite in some regions of Turkey (3,4). Although surgery, chemotherapy, radiotherapy, and combinations thereof play an important role in the treatment of MPM patients, the median survival of patients treated for MPM is dismal, at only 6–18 months (5–7). Despite advances in modern systemic chemotherapy using the combination of pemetrexed and cisplatin, long-term survival in patients with MPM remains limited (8). Therefore, more specific, effective, and less toxic therapies are needed. Research into the molecular pathways of MPM has led to novel targeted strategies that inhibit specific key molecules in tumor growth and progression.

Epidermal growth factor receptor (EGFR) is a tyrosinekinase (TK) receptor involved in cell death and proliferation, cell motility, angiogenesis, and extracellular matrix composition (9). EGFR is overexpressed in many human malignancies, including lung, head and neck, colorectal, and breast cancers, where it is variably associated with patient prognosis (10,11). EGFR is reported to be overexpressed in 44–97% of MPM patients, as determined by various immunohistochemical studies with variability in outcomes (12–15). In a recent study, Destro *et al* demonstrated that both the immunohistochemical expression and corresponding mRNA levels of EGFR were higher in tumor specimens than in normal pleural samples (12). These data confirmed those of a previous study suggesting that EGFR could play an important role in the oncogenic phenotype of MPM disease (9).

Two types of EGFR inhibitors have been developed: small molecule EGFR tyrosine kinase inhibitors (TKIs) (16,17) and monoclonal antibodies directed against the extracellular domain of EGFR (18–20). Gefitinib, a quinazoline derivative, is the first TKI developed that specifically inhibits the activation of EGFR TK through competitive binding to the ATP-binding domain of the receptor. Gefitinib has been shown to be effective in preclinical studies and clinical trials, and it received approval for use in Japan in patients with advanced non-small cell lung cancer refractory to chemotherapy in July 2002. Subsequently, it has gained approval in over 30 countries, including the United States. Gefitinib reduced the proliferation of MPM cells by inhibiting the EGFR signaling pathway *in vitro*(9); however, the clinical study revealed that gefitinib was not active in MPM patients (21). The same is true of erlotinib (14). These disappointing results for EGFR TK inhibitors have led to increased interest in monoclonal antibodies directed against EGFR, because these 2 classes of agents may have substantially different mechanisms of action.

Cetuximab is a chimeric mouse-human antibody directed against the extracellular domain of EGFR (22), thereby inhibiting the binding of activating ligands to the receptor. Consequently, cetuximab inhibits ligand-dependent activation of the EGFR and inhibits the downstream pathways that cause cell cycle progression, cell growth, and angiogenesis. In addition, the binding of cetuximab initiates EGFR internalization and degradation that leads to signal termination (23–25). In addition to these direct inhibitory effects to EGFR signaling, cetuximab potentially provokes immunologic antitumor effects called antibody-dependent cellular cytotoxicity (ADCC). This effect takes place in the presence of the host effector system, such as natural killer (NK) cells, because cetuximab has a human IgG1 backbone. Recently, we and others showed that this ADCC activity is crucial for the antitumor effects of cetuximab (26–28). Because this immunological mechanism is not activated by TKIs, cetuximab is expected to have more potent antitumor activities against MPM than TKIs, especially *in vivo*. However, no published *in vitro* or *in vivo* studies have focused on the effect of cetuximab against MPM cells, particularly with respect to ADCC activity.

In the present study, we investigated the biologic activity of cetuximab against a panel of MPM cells with respect to ADCC activity and the survival effects of intrathoracic treatment using an orthotopic implantation mouse model that reproduces the clinical behavior and therapeutic responsiveness of MPM in humans.

## Materials and methods

### Cell lines and cell culture

Five MPM cell lines (EHMES-1, MSTO-211H, H2052, EHMES-10 and H28) and an epidermoid carcinoma cell line (A431) were used in this study. MSTO-211H, H2052, H28 and A431 were purchased from American Type Culture Collection (ATCC, Manassas, VA, USA). The other lines (EHMES-1, EHMES-10) were established from the pleural effusion of a patient with MPM at Ehime University (Ehime, Japan). All cell lines were maintained in RPMI-1640 supplemented with 10% FCS, 50 U/ml penicillin, 50 U/ml streptomycin and 2.05 mmol/l glutamine. The cells were incubated at 37°C in 5% CO_2_.

### Monoclonal antibody

Cetuximab was obtained from Bristol-Myers Squibb (New York, NY, USA). Rituximab, used as a control antibody, was obtained from Chugai Pharmaceutical (Tokyo, Japan). Anti-EGF receptor antibody (clone 528) for flow cytometry was obtained from Santa Cruz Biotechnology (Santa Cruz, CA, USA). Anti-EGF receptor antibody (clone 31G7) for immunohistochemical analysis was obtained from Zymed (South San Francisco, CA, USA).

### Flow cytometric analysis

Cell surface EGFR expression of MPM cell lines was examined by flow cytometry (Becton-Dickinson, Franklin Lakes, NJ, USA) using a monoclonal antibody (clone 528). To determine the absolute number of antibody-binding sites per cell, we carried out a quantitative flow cytometric analysis using Dako QIFIKIT (DakoCytomation, Copenhagen, Denmark). Briefly, 1×10^4^ cells were incubated for 1 h at 4°C with 0.4 μg of the primary antibody or the isotype-control IgG2a antibody (Sigma-Aldrich, St. Louis, MO, USA) in phosphate-buffered saline (PBS) containing 1% bovine serum albumin (BSA) and 0.01% sodium azide. After washing thrice with PBS, cells were incubated for 1 h with FITC-conjugated anti-mouse IgG (DakoCytomation) at 4°C. Similar to samples labeled with FITC-conjugated anti-mouse IgG from this kit, standard beads coated with a known amount of mouse IgG molecules were labeled with this secondary antibody. The labeled samples were washed thrice with PBS and analyzed using FACScan flow cytometer (Becton Dickinson). The number of antibody binding sites per cell was calculated by comparing the mean fluorescent intensity (MFI) value of the labeled cells with a calibration curve obtained by regression analysis of the MFI values of the standard beads.

### Growth inhibition assay

Cell viability was assessed using the 2-(2-methoxy-4-nitrophenyl)-3-(4-nitrophenyl)-5-(2,4-disulphophenyl)2H-tetrazolium monosodium salt (WST-8) assay (Dojindo, Kumamoto, Japan). Cells were plated at 3×10^4^ cells/well in triplicate in 96-well plates in complete medium. Following an overnight incubation, cetuximab (0-1,000 μg/ml) was added in varying concentrations and incubated. After 72 h, WST-8 solution (Dojindo) was added to each well, followed by incubation for 4 h at 37°C, and absorbance was measured using a Model 680 microplate reader (Bio-Rad Laboratories, Hercules, CA, USA) at test and reference wavelengths of 450 and 655 nm, respectively. Cell viability was calculated by dividing the mean absorbance of wells containing treated cells by those of control wells with untreated cells. The concentration of cetuximab resulting in 50% growth inhibition (IC_50_) was calculated. All experiments were done at least in triplicate and repeated at least 3 times.

### Isolation of peripheral blood mononuclear cells (PBMCs) and interleukin-2 (IL-2) treatment

PBMCs were isolated from heparinized peripheral blood by lymphocyte-separation-medium (MP Biomedicals, Irvine, CA, USA) density gradient centrifugation. To investigate the effect of IL-2 (Sigma-Aldrich) on ADCC activity, PBMCs (10^6^ cells/ml) were pre-incubated at 37°C for up to 18 h before cytotoxic assay in the presence of IL-2 (30 IU/ml) (29–31). Blood samples were collected at Tottori University in accordance with the Tottori University Review Board, and the healthy individuals provided written informed consent.

### Test for ADCC and NK activity

After the target MPM cells were labeled with 100 μCi ^51^Cr (PerkinElmer Life and Analytical Sciences, Boston, MA, USA) for 60 min, target cells (10^4^/well) and effector cells at various effector:target (E/T) ratios were co-incubated in 200 μl of DMEM or RPMI-1640 in a 96-well U-bottomed plate in triplicate for 4 h at 37°C with 0.5 μg/ml of cetuximab (Bristol-Myers Squibb) or control antibody, rituximab (Chugai Pharmaceutical). Next, the amount of radioactivity in the supernatant liquid was measured by a gamma counter. The percentage of specific cytolysis was calculated as previously described (27). ADCC activity was calculated as the percentage of lysis in the presence of cetuximab minus the percentage of lysis in the presence of control antibody that is attributed to NK activity.

### Immunohistochemical analysis

**Figure 1.**
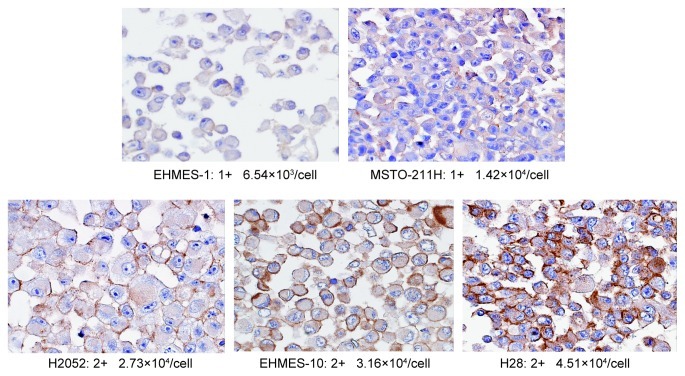
Immunohistochemical staining for EGFR expression. Representative EGFR immunohistochemistry scoring in 5 malignant pleural mesothelioma cell lines. The immunohistochemical score and the number of EGFR molecules/cell are also indicated (×400 magnification).

Paraffin-embedded cell blocks were prepared from each MPM cell lines, which were fixed in 4% paraformaldehyde. Tissue sections (3 μm) were de-waxed in xylene, rehydrated through a graded series of ethanol solutions, rinsed in distilled water for 5 min, and then immersed in 0.6% hydrogen peroxide in methanol for 30 min to block endogenous peroxidase. For antigen retrieval, the sections then were microwaved in 0.01 mol/l of sodium citrate-buffered saline, pH 6.0, for 20 min at 92°C using a Microwave Processor model MI-77 (Azumaya, Tokyo, Japan). After rinsing in PBS for 5 min, the slides were pre-blocked with 10% normal rabbit serum at room temperature for 20 min and incubated at 4°C overnight with the primary antibody, anti-EGF receptor antibody (clone 31G7) (Zymed). The immunoreaction was visualized with 3.3′-diaminobenzidine and 100 μl of hydrogen peroxidase in 0.05 M Tris-HCl buffer, pH 7.6. Finally, the slides were counterstained with a 0.1% hematoxylin solution. The staining results were measured semiquantitatively on a scale of 0, 1+, 2+ and 3+ as follows: 0, no membranous staining in any of the cells; 1+, weak intensity membranous and cytoplasmic staining of nearly equal intensity; 2+, moderate to strong intensity staining predominantly in the membranes; and 3+, strong intensity staining clearly localized to the cell membranes. Representative examples of 0, 1+, 2+ and 3+ IHC staining for EGFR are demonstrated in Fig. 1. We performed the staining for the each cell line 3 times, and the intensity was evaluated by 2 independent pathologists.

### Animals

Male C.B-17 SCID mice (5 weeks) were obtained from CLEA Japan (Osaka, Japan) and maintained under specific pathogen-free conditions throughout the study. Experiments were carried out in accordance with the guidelines established by the Tottori University Committee on Animal Care and Use.

### Orthotopic implantation model

The cultured MSTO-211H cells were harvested by pipetting. The cells were washed 3 times and resuspended in Ca^2+^- and Mg^2+^-free PBS. For orthotopic implantation, SCID mice were anesthetized with ether and had their right chest wall shaved. After sterilization of the chest wall with 70% ethanol, the right chest skin and subcutaneous tissue was cut, and the parietal pleura was exposed. Thereafter, the tumor cells (10^6^/100 μl PBS) were injected into the thoracic cavity of SCID mice using a 27G needle as described previously (32). Finally, the incisions were sutured to close the wound. The mice were treated with cetuximab (0.05 mg/mouse i.t.) or in combination with IL-2 (30 IU/ml i.t.) using the same methods on day 7, and sacrificed on day 21 to evaluate tumor development. The pleura-disseminated tumors were inspected macroscopically.

### Area measurements

The intrathoracic tumor area was manually defined on intrathoracic pictures, and was measured with the image analysis software program Scion Image for Windows (PC version of NIH Image).

### Statistics

The statistical comparison between the 2 groups was analyzed using Student’s t-test. The survival times of SCID mice bearing MSTO-211H cells was determined using the Kaplan-Meier estimation (PRISM for Windows; GraphPad Software, La Jolla, CA, USA).

## Results

### Analysis of EGFR expression in MPM cell lines using flow cytometry and IHC

We first examined the expression of EGFR in 5 MPM cell lines. A431, an epidermoid carcinoma cell line, was used as a positive control for EGFR expression in most studies, since it has been reported to express high levels of EGFR (33,34). We measured the number of EGFRs on each MPM cell line by quantitative flow cytometric analysis (Dako QIFIKIT) (35) and compared them to the evaluation by immunohistochemistry (IHC) (scored from 0 to 3+). As shown in Table I, the level of EGFR expression in each MPM cell line, in ascending order, is as follows: EHMES-1, MSTO-211H, H2052, EHMES-10 and H28. As assessed using IHC, 2 cell lines (EHMES-1 and MSTO-211H), which express a low number of EGFRs (ranging from 6.54×10^3^ to 1.42×10^4^/cell), were stained and scored as 1+. The other 3 cell lines of MPM, which expressed moderate numbers of EGFR (ranging from 2.73×10^4^ to 4.51×10^4^/cell), were scored as 2+. The positive control cell line A431, expressing a large number of EGFRs (3.51×10^6^/cell) scored 3+ (data not shown) (Fig. 1). These results indicated a good correlation between the number of EGFR molecules on the cells and their EGFR status as estimated by IHC.

### Direct effects of cetuximab on growth inhibition in MPM cells

**Figure 2.**
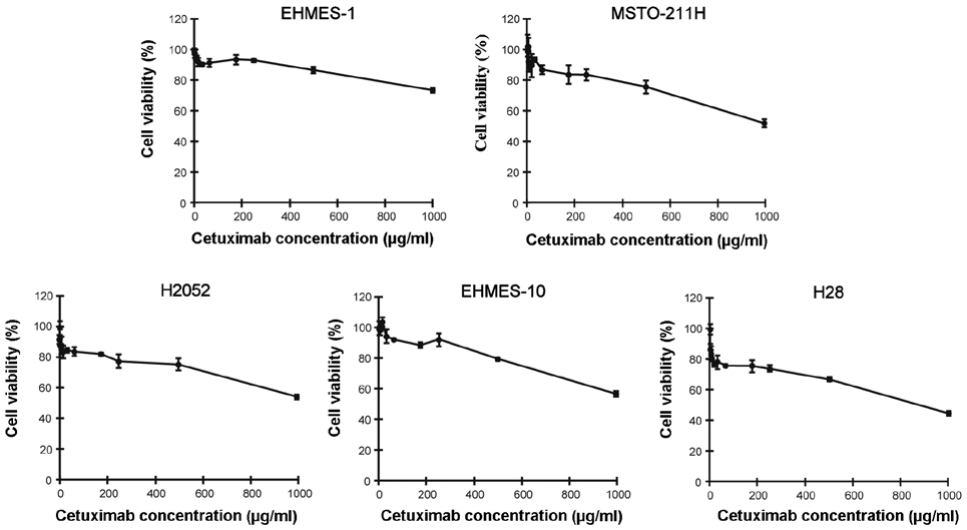
Direct effects of cetuximab on growth inhibition. Malignant pleural mesothelioma cell lines were treated with indicated concentration of cetuximab (0-1,000 μg/ml). Cell proliferation was measured by WST-8 assay after 72 h of continuous drug exposure.

We next examined the effect of cetuximab against the proliferation of MPM cells using the WST-8 assay, which is a modified MTT assay. We found that all MPM cell lines were completely resistant to cetuximab treatment irrespective of the surface amount of EGFR (Fig. 2). These data suggest that direct growth inhibitory effects would not be expected in the anti-MPM action of cetuximab.

### Cetuximab-mediated cytotoxicity against MSTO-211H cells by healthy human PBMCs

**Figure 3.**
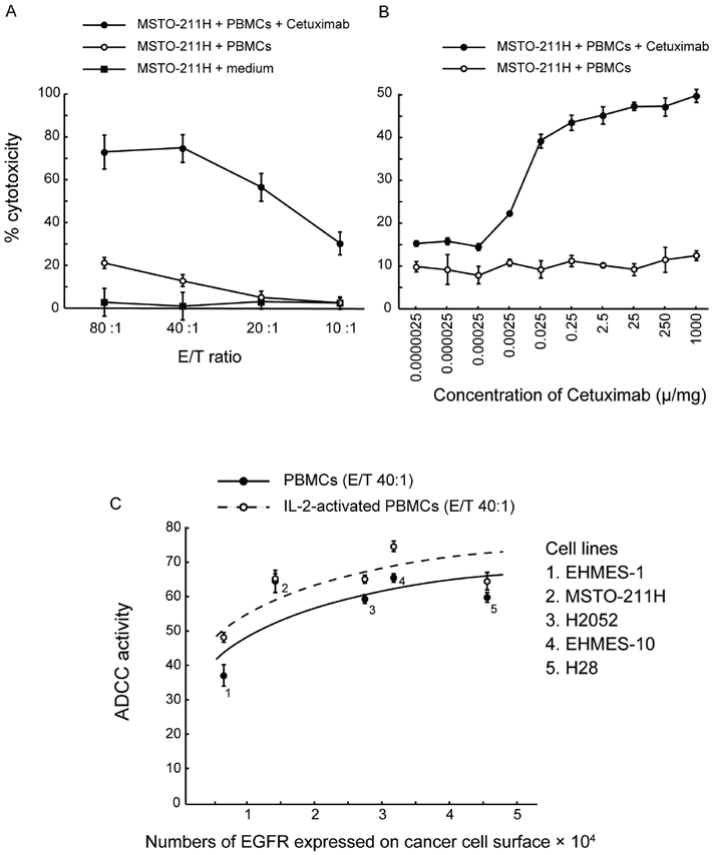
Cytotoxicity against a malignant pleural mesothelioma cell line mediated by cetuximab. (A) Cetuximab mediates cytotoxicity against the EGFR-expressing MSTO-211H mesothelioma cell line. Healthy human PBMCs, using 4 different E/T ratios, were tested for cytotoxicity in the presence or absence of cetuximab (2.5 μg/ml). The y-axis reveals cytotoxicity as determined by a 4-h ^51^Cr release assays. (B) Concentration-dependent curve of cetuximab-dependent ADCC activity and NK activity against MSTO-211H cells by healthy human PBMCs. MSTO-211H cells were incubated with PBMCs at an E/T ratio of 20:1 along with or without indicated concentrations of cetuximab (0.0000025-1,000 μg/ml). Data are representative of 3 independent experiments. Points, mean of a triplicate experiment; bars, SD. (C) Correlation between EGFR expression levels of target malignant mesothelioma cell lines and cetuximab-mediated ADCC activity. The x-axis indicates the number of EGFR molecules expressed on the surface of the cancer cells. The y-axis represents the ADCC activity of cetuximab (0.25 mg/ml) as determined by a 4-h ^51^Cr release assay. Healthy human PBMCs were incubated with or without IL-2 (30 IU/ml) at an E/T ratio of 40:1 for 18 h and tested for cetuximab-mediated ADCC activity against various ^51^Cr-labeled cell lines. Data are representative of 5 independent experiments. The malignant mesothelioma cell lines used are indicated. Points, mean of a triplicate experiment; bars, SD.

To test whether cetuximab induces ADCC activity against MPM cell lines, we performed a 4-h ^51^Cr release assay of MSTO-211H cells that weakly express EGFR using human PBMCs at various E/T ratios (Fig. 3A). While low levels of cytolysis of MSTO-211H cells were induced by PBMCs at the higher E/T ratios of 80:1 and 40:1 in the absence of cetuximab (known as NK activity), the lytic activity of PBMCs increased significantly in the presence of cetuximab at both E/T ratios. There was no significant increase in lytic activity in the presence of the control antibody, rituximab (data not shown). These data suggest that cetuximab was capable of inducing ADCC activity efficiently, even against MPM cells that weakly express EGFR.

Next, to identify the optimal cetuximab concentration for ADCC activity, we determined the ADCC activity with increasing concentrations of cetuximab, ranging from 2.5×10^−6^ to 1,000 mg/ml at an E/T ratio of 20:1. As shown in Fig. 3B, cetuximab-mediated ADCC activity against MSTO-211H cells was already detectable at a concentration of 2.5×10^−3^ mg/ml and was saturated at 0.25 mg/ml. These data indicate that a cetuximab concentration in excess of 0.25 mg/ml was sufficient for maximum ADCC activity. We used this concentration of cetuximab for the subsequent assays.

### Cetuximab-mediated ADCC activity against MPM cell lines with various EGFR expression levels

To evaluate the correlation between the ADCC activity induced by cetuximab and EGFR expression levels on target MPM cells, we determined the ADCC activity in MPM cell lines with various EGFR expression levels at an E/T ratio of 40:1 in the presence of the optimal dose of cetuximab (0.25 mg/ml). As shown in Fig. 3C, the ADCC activity correlated logarithmically with the number of EGFR molecules expressed on the MPM cell surface. Near-maximum ADCC activity was observed in MSTO-211H cells, which have small numbers of EGFRs and scored 1+ by IHC. ADCC activity did not increase in cells with higher EGFR expression.

In addition, as IL-2 is known to activate PBMCs, we tested the effects of overnight treatment of PBMCs with IL-2 on cetuximab-mediated ADCC activity. Low doses of IL-2 increased ADCC activity in all cell lines, regardless of EGFR expression level (Fig. 3C). These data suggest that the very weak EGFR expression in MPM cells is enough to mediate ADCC activity and that IL-2 is capable of enhancing this activity.

### Effect of cetuximab and IL-2 on SCID mice bearing MSTO-211H cells

**Figure 4.**
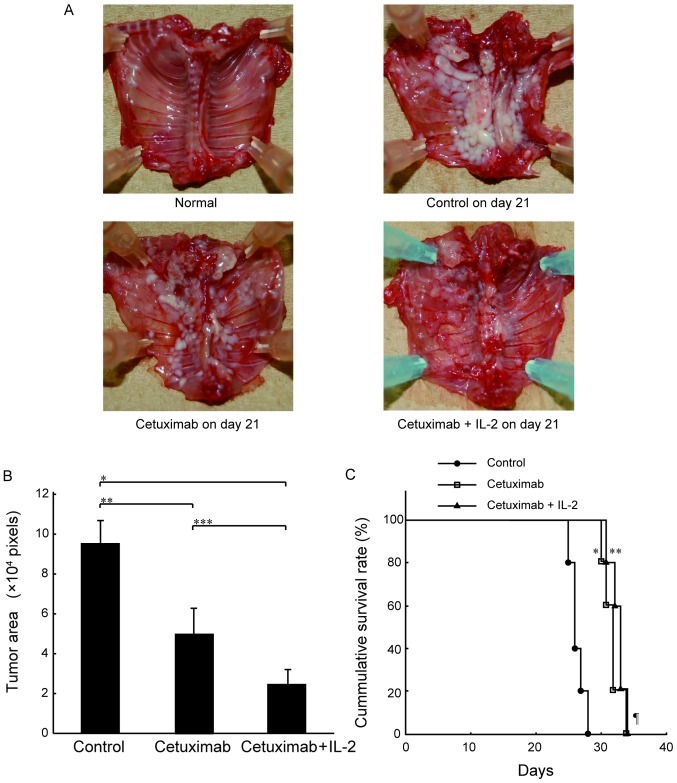
Effect of cetuximab alone and in combination with IL-2 on SCID mice bearing MSTO-211H cells. Mice were treated with cetuximab (0.05 mg/mouse i.t. on day 7) or in combination with IL-2 (30 IU/ml i.t. on day 7). (A) Representative intrathoracic pictures of SCID mice bearing MSTO-211H cells on day 21. The MSTO-211H cells produced small nodular tumors intrathoracic cavity. (B) Tumor area of the mice treated with cetuximab or in combination with IL-2 on day 21. Columns, mean pixels from 5 independent animals; bars, SD. ^*^P<0.001 compared to the control group; ^**^P<0.001 compared to the control group; ^***^P<0.01 compared to the cetuximab monotherapy group. (C) Survival time of SCID mice bearing MSTO-211H cells. Kaplan-Meier survival curves are displayed. The mice (N=5/group) were treated with cetuximab (0.05 mg/mouse i.t. on day 7) or in combination with IL-2 (30 IU/ml i.t. on day 7). Intrathoracic administration of cetuximab significantly prolonged the survival time of the mice compared to that of the control group. ^*^P<0.01 compared to the control group; ^**^P<0.01 compared to the control group; ¶, P=0.28 compared to the cetuximab monotherapy group.

To test the antitumor effects of cetuximab *in vivo*, we used an orthotopic implantation mouse model as a clinically relevant animal model. In this model, cells from the mesothelioma cell line MSTO-211H were implanted into the thoracic cavity of SCID mice, which possess robust NK cell activity (36). The implanted mice were treated with cetuximab by direct administration into the thoracic cavity with and without IL-2 on day 7, and then sacrificed on day 21 as described in Materials and methods. To determine the optimal dose of cetuximab for the treatment of SCID mice bearing MSTO-211H, we first determined the survival times of the mice using various amounts of cetuximab (0.5 mg/mouse, 0.05 mg/mouse and 0.005 mg/mouse i.t. on day 7). This preliminary experiment showed that there was no statistically significant difference in survival time between the mice (data not shown). In addition, in a separate study that evaluated the pharmacokinetics of cetuximab in nude mice bearing human colon carcinoma xenografts, the efficacious range for antitumor activity was demonstrated to be 0.04-1 mg/mouse (37). We therefore administered cetuximab at a dose of 0.05 mg/mouse in our subsequent *in vivo* experiments to ensure biological activity yet minimize side effects. As shown in Fig. 4A, cetuximab inhibited intrathoracic mesothelioma growth in the mice, and this inhibition was markedly enhanced by IL-2 co-administration. This inhibitory effect of cetuximab alone and its enhancement by the addition of IL-2 was confirmed by quantitative measurements of the tumor area using Scion Image Software (Fig. 4B). Furthermore, intrathoracic administration of cetuximab significantly prolonged the survival of the mice, and the combination of cetuximab with IL-2 tended to improve survival (Fig. 4C). These results suggest that cetuximab exerts antitumor effects against MPM cells in the presence of the mouse effector system, and that ADCC activity is highly involved in this effect.

## Discussion

In the present study, we evaluated cetuximab as a novel molecular targeting agent for MPM. We found that cetuximab induces potent ADCC activity but not growth inhibition against MPM cell lines. Cetuximab-induced ADCC activity has several characteristics that are relevant to clinical therapeutic applications. First, low concentrations of cetuximab are sufficient to induce maximum ADCC activity. Second, the low EGFR expression levels on MPM cells, which are scored as 1+ by IHC, could be sufficient for maximum ADCC activity mediated by cetuximab. Third, *ex vivo* IL-2 treatment of PBMCs can enhance cetuximab-mediated ADCC activity against MPM cell lines. Finally, intrathoracic administration of cetuximab in an orthotopic implantation mouse model significantly inhibited tumor growth and prolonged the survival time in the presence of the mouse effector system. These data indicate the important role of ADCC activity in the mechanism of action of cetuximab against cancer cells and underscore the promising potential of cetuximab as a new class of therapeutic agent for use against MPM.

In this study, we examined the correlation between the number of EGFRs on the cell surface and cetuximab-induced ADCC activity in MPM cells and found that there is a logarithmic relationship between them. This finding is in agreement with our previous observations and those reported by others in relation to other cancers. The ADCC activities of trastuzumab (38), anti-Ep-CAM antibody (39) and cetuximab (27) have been reported to weakly correlate with the logarithm of the number of target cell surface antigens in breast or lung cancer cells. The correlation observed in this study indicates that low EGFR expression levels could be sufficient for maximum ADCC activity of cetuximab against MPM cells and that an increase in the expression level of EGFR has no obvious effect on ADCC activity.

Our results indicate the possible usefulness of EGFR IHC as a predictive marker of the effectiveness of cetuximab-mediated ADCC activity against MPM cells. We demonstrated that the demarcation point of the EGFR expression level to achieve maximum ADCC activity is between EHMES-1 (6.54×10^3^ EGFR molecules/cell) and MSTO-211H (1.42×10^4^ EGFR molecules/cell), both of which are scored as 1+ by IHC. Therefore, near-maximum ADCC activity could be expected as long as the cells are stained by IHC, independent of the strength of the staining. This feature could circumvent a common weak point of IHC, as the semi-quantitative (40) nature of IHC makes it prone to inter-observer scoring error. In addition, IHC is superior to other methods for measuring EGFR levels in clinical specimens, such as a ligand binding assay (41) and quantitative flow cytometry (42), because it does not require isolation of cells from fresh tissue or special equipment. Therefore, IHC might be useful if it is scored simply as negative or positive when assessing a tumor sample for predicting the effectiveness of the ADCC activity of cetuximab.

We have demonstrated that cetuximab-mediated ADCC activity against MPM cell lines is enhanced in response to IL-2. This lymphokine is normally produced by T-lymphocytes and augments the function of effector cells, such as B cells, NK cells, T cells and monocytes (43). The combination of IL-2 and a therapeutic monoclonal antibody has been explored extensively in the case of rituximab (44,45) and trastuzumab (46–49) and has been reported to enhance ADCC activity *in vitro*(50) or *in vivo* using mouse xenograft models (51). Based on these fundamental studies, several preclinical trials of these combination therapies have been conducted, including rituximab for B cell non-Hodgkin’s lymphoma (52) and trastuzumab for HER2-overexpressing cancer (53,54). Therefore, our observation that IL-2 enhances cetuximab-meditated ADCC activity against MPM cell lines might lend support to the future concurrent use of cetuximab and IL-2 in patients with MPM.

In our study, we showed that intrathoracic administration of cetuximab significantly inhibited tumor growth and prolonged the survival of mice. Several lines of evidences suggest that the anti-MPM effects of cetuximab *in vivo* are dependent on ADCC activity. First, murine spleen cells derived from SCID mice have been reported to induce ADCC activity against melanoma cells treated with cetuximab, though the effect is not as potent as that by parental mouse monoclonal antibodies against EGFR (26). In addition, several reports have described that the mouse effector system can induce the ADCC activity of human IgG1 antibody (55,56). Taken together, it can be concluded that mouse effector cells can bind to the Fc portion of human IgG to some extent, exerting some level of ADCC activity, if not its full activity. Second, our preliminary observation that there was no difference in survival times between mice treated with different amounts of cetuximab is strikingly similar to the dose-effector relationship of cetuximab-induced ADCC activity shown *in vitro*; that is, a cetuximab concentration in excess of 0.25 mg/ml is sufficient for maximum ADCC activity *in vitro*, and higher concentrations have no effect on the activity. Third, we used C.B-17 SCID mice, which lack mature T- and B-lymphocytes but possess robust NK cell activity (36). We have shown in a previous report that only NK cells are major effectors of cetuximab-mediated ADCC activity that is augmented by IL-2 (27). In parallel, IL-2 co-administration with cetuximab significantly inhibited MPM tumor growth in our model. Considering these data, we believe that our successful treatment of MPM in the SCID mouse model reflects cetuximab-induced ADCC activity and that future efforts to enhance this ADCC activity with effective adjuvants, such as cytokines (57), would be of vital importance.

This is the first study to investigate intrathoracic treatment by cetuximab for MPM. Because mesothelioma tends to remain localized in the pleural cavity for a long time, the development of local treatments would be promising. For this purpose, the orthotopic mouse model of MPM would be ideal for the evaluation of cetuximab, because MPM cells mimic the clinical behavior and progression of human MPM in this model (32). Local treatment with antitumor drugs offers a theoretical advantage, because the tumor is exposed directly to higher drug concentrations, while a lower incidence of toxic side effect can be expected. To date, several local treatments have been reported as successful in combination with other therapeutic modalities, such as surgery. These local treatments include intrathoracic chemotherapy (58), chemohyperthermia (59), and intraoperative photodynamic therapy (IPDT) (60). Our proposed combination therapy using cetuximab with IL-2 is preferable for the local treatment of MPM, because IL-2 causes serious side effects such as vascular leakage syndrome and systemic immuno-suppression if administered systemically (61). Local treatment may not be expected to cure MPM patients, but the improved local control of MPM in the thoracic cavity in combination with systemic treatment could offer potential benefits for MPM patients.

In this study, cetuximab treatment significantly inhibited intrathoracic MPM tumor growth, and the addition of IL-2 enhanced this activity. However, contrary to our expectations, the combined use of cetuximab and IL-2 did not improve survival of the mice compared to cetuximab alone. There are 2 possible explanations for this result. First, the number of mice we used in each group (N=5) was not enough to detect the difference. Second, the effects on tumor growth might not directly affect the survival of the mice due to distant metastasis or the side effects of IL-2, such as cardiac failure (62). Therefore, further study is warranted to determine the cause of death in these mice and to explore more effective and less toxic combination-use of IL-2.

In conclusion, cetuximab induces ADCC activity against EGFR-expressing MPM cell lines. Near-maximum ADCC activity was observed in cells with very weak EGFR expression levels, which was detectable as faint IHC staining. ADCC activity is enhanced at any EGFR expression level in the presence of low doses of IL-2. Intrathoracic administration of cetuximab in SCID mice bearing MSTO-211H cells significantly inhibited tumor growth and prolonged survival of the mice. These observations suggest the possible use of cetuximab as a novel and effective therapeutic agent that could be used in combination therapies for patients with MPM.

## Figures and Tables

**Table I.** EGFR expression analysis by quantitative flow cytometry and IHC in malignant pleural mesothelioma cell lines.

Cell lines	EGFR expression (nos. of EGFR/cells)	Immunohistochemical score
EHMES-1	6.54×10^3^	1+
MSTO-211H	1.42×10^4^	1+
H2052	2.73×10^4^	2+
EHMES-10	3.16×10^4^	2+
H28	4.51×10^4^	2+

## References

[1] Greillier L, Astoul P (2008). Mesothelioma and asbestos-related pleural diseases. Respiration.

[2] Wagner JC, Sleggs CA, Marchand P (1960). Diffuse pleural mesothelioma and asbestos exposure in the North Western Cape Province. Br J Ind Med.

[3] Dumortier P, Coplu L, de Maertelaer V, Emri S, Baris I, De Vuyst P (1998). Assessment of environmental asbestos exposure in Turkey by bronchoalveolar lavage. Am J Respir Crit Care Med.

[4] Baris B, Demir AU, Shehu V, Karakoca Y, Kisacik G, Baris YI (1996). Environmental fibrous zeolite (erionite) exposure and malignant tumors other than mesothelioma. J Environ Pathol Toxicol Oncol.

[5] Ruffie P, Feld R, Minkin S (1989). Diffuse malignant mesothelioma of the pleura in Ontario and Quebec: a retrospective study of 332 patients. J Clin Oncol.

[6] Pass HI, Kranda K, Temeck BK, Feuerstein I, Steinberg SM (1997). Surgically debulked malignant pleural mesothelioma: results and prognostic factors. Ann Surg Oncol.

[7] Rusch VW, Piantadosi S, Holmes EC (1991). The role of extra-pleural pneumonectomy in malignant pleural mesothelioma. A Lung Cancer Study Group trial. J Thorac Cardiovasc Surg.

[8] Vogelzang NJ, Rusthoven JJ, Symanowski J (2003). Phase III study of pemetrexed in combination with cisplatin versus cisplatin alone in patients with malignant pleural mesothelioma. J Clin Oncol.

[9] Janne PA, Taffaro ML, Salgia R, Johnson BE (2002). Inhibition of epidermal growth factor receptor signaling in malignant pleural mesothelioma. Cancer Res.

[10] Arteaga CL (2002). Epidermal growth factor receptor dependence in human tumors: more than just expression?. Oncologist.

[11] Brabender J, Danenberg KD, Metzger R (2001). Epidermal growth factor receptor and HER2-neu mRNA expression in non-small cell lung cancer is correlated with survival. Clin Cancer Res.

[12] Destro A, Ceresoli GL, Falleni M (2006). EGFR overexpression in malignant pleural mesothelioma. An immunohistochemical and molecular study with clinico-pathological correlations. Lung Cancer.

[13] Okuda K, Sasaki H, Kawano O (2008). Epidermal growth factor receptor gene mutation, amplification and protein expression in malignant pleural mesothelioma. J Cancer Res Clin Oncol.

[14] Garland LL, Rankin C, Gandara DR (2007). Phase II study of erlotinib in patients with malignant pleural mesothelioma: a Southwest Oncology Group Study. J Clin Oncol.

[15] Agarwal V, Lind MJ, Cawkwell L (2010). Targeted epidermal growth factor receptor therapy in malignant pleural mesothelioma: where do we stand?. Cancer Treat Rev.

[16] Kim ES, Hirsh V, Mok T (2008). Gefitinib versus docetaxel in previously treated non-small-cell lung cancer (INTEREST): a randomised phase III trial. Lancet.

[17] Shepherd FA, Rodrigues Pereira J, Ciuleanu T (2005). Erlotinib in previously treated non-small-cell lung cancer. N Engl J Med.

[18] Albanell J, Codony-Servat J, Rojo F (2001). Activated extracellular signal-regulated kinases: association with epidermal growth factor receptor/transforming growth factor alpha expression in head and neck squamous carcinoma and inhibition by anti-epidermal growth factor receptor treatments. Cancer Res.

[19] Goldstein NI, Prewett M, Zuklys K, Rockwell P, Mendelsohn J (1995). Biological efficacy of a chimeric antibody to the epidermal growth factor receptor in a human tumor xenograft model. Clin Cancer Res.

[20] Jonker DJ, O’Callaghan CJ, Karapetis CS (2007). Cetuximab for the treatment of colorectal cancer. N Engl J Med.

[21] Govindan R, Kratzke RA, Herndon JE (2005). Gefitinib in patients with malignant mesothelioma: a phase II study by the Cancer and Leukemia Group B. Clin Cancer Res.

[22] Li S, Schmitz KR, Jeffrey PD, Wiltzius JJ, Kussie P, Ferguson KM (2005). Structural basis for inhibition of the epidermal growth factor receptor by cetuximab. Cancer Cell.

[23] Sato JD, Kawamoto T, Le AD, Mendelsohn J, Polikoff J, Sato GH (1983). Biological effects in vitro of monoclonal antibodies to human epidermal growth factor receptors. Mol Biol Med.

[24] Gill GN, Kawamoto T, Cochet C (1984). Monoclonal anti-epidermal growth factor receptor antibodies which are inhibitors of epidermal growth factor binding and antagonists of epidermal growth factor binding and antagonists of epidermal growth factor-stimulated tyrosine protein kinase activity. J Biol Chem.

[25] Kawamoto T, Sato JD, Le A, Polikoff J, Sato GH, Mendelsohn J (1983). Growth stimulation of A431 cells by epidermal growth factor: identification of high-affinity receptors for epidermal growth factor by an anti-receptor monoclonal antibody. Proc Natl Acad Sci USA.

[26] Naramura M, Gillies SD, Mendelsohn J, Reisfeld RA, Mueller BM (1993). Therapeutic potential of chimeric and murine anti-(epidermal growth factor receptor) antibodies in a metastasis model for human melanoma. Cancer Immunol Immunother.

[27] Kurai J, Chikumi H, Hashimoto K (2007). Antibody-dependent cellular cytotoxicity mediated by cetuximab against lung cancer cell lines. Clin Cancer Res.

[28] Kimura H, Sakai K, Arao T, Shimoyama T, Tamura T, Nishio K (2007). Antibody-dependent cellular cytotoxicity of cetuximab against tumor cells with wild-type or mutant epidermal growth factor receptor. Cancer Sci.

[29] Henney CS, Kuribayashi K, Kern DE, Gillis S (1981). Interleukin-2 augments natural killer cell activity. Nature.

[30] Liu Z, Lee FT, Hanai N (2002). Cytokine enhancement of in vitro antibody-dependent cellular cytotoxicity mediated by chimeric anti-GD3 monoclonal antibody KM871. Cancer Immun.

[31] Nguyen QH, Roberts RL, Ank BJ, Lin SJ, Lau CK, Stiehm ER (1998). Enhancement of antibody-dependent cellular cytotoxicity of neonatal cells by interleukin-2 (IL-2) and IL-12. Clin Diagn Lab Immunol.

[32] Nakataki E, Yano S, Matsumori Y (2006). Novel orthotopic implantation model of human malignant pleural mesothelioma (EHMES-10 cells) highly expressing vascular endothelial growth factor and its receptor. Cancer Sci.

[33] Todaro GJ, De Larco JE (1978). Growth factors produced by sarcoma virus-transformed cells. Cancer Res.

[34] Wikstrand CJ, McLendon RE, Friedman AH, Bigner DD (1997). Cell surface localization and density of the tumor-associated variant of the epidermal growth factor receptor, EGFRvIII. Cancer Res.

[35] Brockhoff G, Hofstaedter F, Knuechel R (1994). Flow cytometric detection and quantitation of the epidermal growth factor receptor in comparison to Scatchard analysis in human bladder carcinoma cell lines. Cytometry.

[36] Dorshkind K, Pollack SB, Bosma MJ, Phillips RA (1985). Natural killer (NK) cells are present in mice with severe combined immunodeficiency (scid). J Immunol.

[37] Luo FR, Yang Z, Dong H (2005). Correlation of pharmacokinetics with the antitumor activity of Cetuximab in nude mice bearing the GEO human colon carcinoma xenograft. Cancer Chemother Pharmacol.

[38] Niwa R, Sakurada M, Kobayashi Y (2005). Enhanced natural killer cell binding and activation by low-fucose IgG1 antibody results in potent antibody-dependent cellular cytotoxicity induction at lower antigen density. Clin Cancer Res.

[39] Prang N, Preithner S, Brischwein K (2005). Cellular and complement-dependent cytotoxicity of Ep-CAM-specific monoclonal antibody MT201 against breast cancer cell lines. Br J Cancer.

[40] Rallet A, Faroux MJ, Theobald S (1994). Epidermal growth factor receptors in breast cancer: comparison of radioligand and immunocytochemical assays. Anticancer Res.

[41] Klijn JG, Berns PM, Schmitz PI, Foekens JA (1992). The clinical significance of epidermal growth factor receptor (EGF-R) in human breast cancer: a review on 5232 patients. Endocr Rev.

[42] Kimmig R, Pfeiffer D, Landsmann H, Hepp H (1997). Quantitative determination of the epidermal growth factor receptor in cervical cancer and normal cervical epithelium by 2-color flow cytometry: evidence for down-regulation in cervical cancer. Int J Cancer.

[43] Smith KA (1988). Interleukin-2: inception, impact, and implications. Science.

[44] Hooijberg E, Sein JJ, van den Berk PC (1995). Eradication of large human B cell tumors in nude mice with unconjugated CD20 monoclonal antibodies and interleukin 2. Cancer Res.

[45] Golay J, Manganini M, Facchinetti V (2003). Rituximab-mediated antibody-dependent cellular cytotoxicity against neoplastic B cells is stimulated strongly by interleukin-2. Haematologica.

[46] Kubo M, Morisaki T, Kuroki H (2003). Combination of adoptive immunotherapy with Herceptin for patients with HER2-expressing breast cancer. Anticancer Res.

[47] Carson WE, Parihar R, Lindemann MJ (2001). Interleukin-2 enhances the natural killer cell response to Herceptin-coated Her2/neu-positive breast cancer cells. Eur J Immunol.

[48] Kono K, Takahashi A, Ichihara F, Sugai H, Fujii H, Matsumoto Y (2002). Impaired antibody-dependent cellular cytotoxicity mediated by herceptin in patients with gastric cancer. Cancer Res.

[49] Santin AD, Bellone S, Gokden M (2002). Overexpression of HER-2/neu in uterine serous papillary cancer. Clin Cancer Res.

[50] Berinstein N, Levy R (1987). Treatment of a murine B cell lymphoma with monoclonal antibodies and IL-2. J Immunol.

[51] Vuist WM, v Buitenen F, de Rie MA, Hekman A, Rumke P, Melief CJ (1989). Potentiation by interleukin 2 of Burkitt’s lymphoma therapy with anti-pan B (anti-CD19) monoclonal antibodies in a mouse xenotransplantation model. Cancer Res.

[52] Gluck WL, Hurst D, Yuen A (2004). Phase I studies of interleukin (IL)-2 and rituximab in B-cell non-Hodgkin’s lymphoma: IL-2 mediated natural killer cell expansion correlations with clinical response. Clin Cancer Res.

[53] Fleming GF, Meropol NJ, Rosner GL (2002). A phase I trial of escalating doses of trastuzumab combined with daily subcutaneous interleukin 2: report of cancer and leukemia group B 9661. Clin Cancer Res.

[54] Repka T, Chiorean EG, Gay J (2003). Trastuzumab and inter-leukin-2 in HER2-positive metastatic breast cancer: a pilot study. Clin Cancer Res.

[55] Suzuki M, Kato-Nakano M, Kawamoto S (2009). Therapeutic antitumor efficacy of monoclonal antibody against Claudin-4 for pancreatic and ovarian cancers. Cancer Sci.

[56] Lopes de Menezes DE, Denis-Mize K, Tang Y (2007). Recombinant interleukin-2 significantly augments activity of rituximab in human tumor xenograft models of B-cell non-Hodgkin lymphoma. J Immunother.

[57] Roda JM, Joshi T, Butchar JP (2007). The activation of natural killer cell effector functions by cetuximab-coated, epidermal growth factor receptor positive tumor cells is enhanced by cytokines. Clin Cancer Res.

[58] Aziz T, Jilaihawi A, Prakash D (2002). The management of malignant pleural mesothelioma; single centre experience in 10 years. Eur J Cardiothorac Surg.

[59] Monneuse O, Beaujard AC, Guibert B (2003). Long-term results of intrathoracic chemohyperthermia (ITCH) for the treatment of pleural malignancies. Br J Cancer.

[60] Schouwink H, Rutgers ET, van der Sijp J (2001). Intraoperative photodynamic therapy after pleuropneumonectomy in patients with malignant pleural mesothelioma: dose finding and toxicity results. Chest.

[61] Den Otter W, Jacobs JJ, Battermann JJ (2008). Local therapy of cancer with free IL-2. Cancer Immunol Immunother.

[62] Castagneto B, Zai S, Mutti L (2001). Palliative and therapeutic activity of IL-2 immunotherapy in unresectable malignant pleural mesothelioma with pleural effusion: results of a phase II study on 31 consecutive patients. Lung Cancer.

